# Loneliness in early psychosis: a qualitative study exploring the views of mental health practitioners in early intervention services

**DOI:** 10.1186/s12888-021-03138-w

**Published:** 2021-03-06

**Authors:** Theodora Stefanidou, Jingyi Wang, Nicola Morant, Brynmor Lloyd-Evans, Sonia Johnson

**Affiliations:** 1 Division of Psychiatry, University College London, London, UK; 2 Department of Social Medicine, School of Public Health, Fudan University, Shanghai, China; 3 Camden and Islington NHS Foundation Trust, London, UK

**Keywords:** Loneliness, Early psychosis, Mental health practitioners, Qualitative research

## Abstract

**Background:**

Loneliness is an important public health problem with established adverse effects on physical and mental health. Although people with psychosis often experience high levels of loneliness, relatively little is known about the relationship between loneliness and early psychosis. Potential interventions to address loneliness might be easier to implement early in the illness when social networks and social skills may be more intact than at a later stage. We investigated the views of mental health practitioners about the context and causes of loneliness in people with early psychosis, and about potential interventions.

**Methods:**

Semi-structured face-to-face interviews were conducted with mental health practitioners (*n* = 20). Participants were purposively recruited from four early intervention services for first-episode psychosis in the UK. Interviews were transcribed verbatim and thematic analysis was conducted.

**Results:**

Participants believed that the majority of service users with early psychosis experience feelings of loneliness. They often saw socially isolated and disconnected clients and believed them to be lonely, but rarely discussed loneliness explicitly in clinical interactions. A combination of symptoms, stigma and negative sense of self were believed to underpin loneliness. Participants could not identify any specific current interventions delivered by their services for tackling loneliness, but thought some routinely provided interventions, including social groups and psychological treatments, could be helpful. They favoured making a wider range of loneliness interventions available and believed that community agencies beyond mental health services should be involved to make these effective and feasible to deliver. They suggested social participation interventions without an explicit mental health focus as potentially promising and valued a co-produced approach to intervention development.

**Conclusions:**

This study suggests that loneliness is not routinely discussed in early intervention services, and a targeted strategy for tackling it is lacking. Co-produced, individualised community approaches, and interventions that target symptoms, stigma and negative self-schemas might be beneficial in alleviating loneliness for people with early psychosis. Empirical research is needed to develop and test such interventions.

**Supplementary Information:**

The online version contains supplementary material available at 10.1186/s12888-021-03138-w.

## Background

Loneliness is a distressing feeling stemming from the subjectively experienced gap between one’s actual and desired social relations [1]. It relates to perceived social isolation, and is closely associated with the quality of social relationships [2]. Thus, it should be distinguished from quantitative features of social support, such as objective social isolation and social network size [3]. Capturing the multidimensional nature of loneliness, Weiss (1973) [4] classified loneliness into social and emotional dimensions. According to this typology, social loneliness resulted from difficulties with social integration, and emotional loneliness is derived from the absence of a close intimate attachment to another person [5]. Cacioppo et al., 2015 [6] distinguished intimate, relational and collective loneliness, relating to the quality of someone’s intimate relationships, friendships and social relationships, and sense of belonging in society.

Enduring feelings of loneliness predict a range of poor physical and mental health outcomes [2]. In the general population, feelings of loneliness are associated with increased morbidity and mortality rates [7–9]. Loneliness has been associated with increased blood pressure [10], sleep disturbances [11], suicidality [12], depression, anxiety, lower life satisfaction [2, 13–15], and increased risk for Alzheimer’s disease [16, 17].

Research has mainly focused on the impact of loneliness in adolescents and older people but there is accumulating evidence that across the lifespan, people with mental health problems are especially vulnerable to loneliness, and tend to have fewer social contacts than the general population [18–20]. According to the 2010 Australian national psychosis survey, 80% of people with psychosis reported loneliness and identified it as a major challenge in their recovery [21, 22]. These findings are in line with systematic review and meta-analytic data suggesting a significant positive relationship between loneliness and psychotic symptom severity [23, 24].

Additionally, there is growing evidence that high rates of loneliness, lack of a confidant, poor perceived social support and reduced social networks are already evident at first-episode psychosis, with cross-sectional evidence indicating anxiety as one of the pathways whereby loneliness affects psychosis [25, 26]. Although a cross sectional relationship between loneliness and psychotic symptoms has been well established, the causal status of the observed association is yet to be determined [27].

To date, an evidence-based intervention for loneliness in psychosis has not been developed [20, 28, 29]. Some strategies for loneliness have been developed in other populations and are a potential source for approaches to address loneliness among people with psychosis. While cognitive approaches show some promise, no interventions for loneliness have clearly demonstrated effectiveness, and only few have been tested with people with psychosis [29, 30]. Moreover, increasing social contact in itself might not be enough to reduce loneliness and improve personal and health outcomes in early psychosis [31]. Thus, more research is needed to investigate a range of different types of support aiming at enhancing both the quality and quantity of people’s social relationships [28].

Hence, loneliness is frequent among people with psychosis, and would be expected to have effects on physical and mental health at least as harmful as those documented in the population as a whole. There is a case for developing and testing targeted loneliness interventions in this population, a need that may be more readily addressed early in illness when social networks and social skills may be more intact than at a later stage. Theory building is one of the key initial stages in intervention design, and clinicians have one of the significant perspectives on the potential mechanisms and characteristics for an effective intervention, and on how and by whom it could be delivered. The current study contributes to the foundations for intervention development by exploring this perspective.

We sought the views of practitioners in specialist Early Intervention Services (EISs), which have been implemented internationally and, in England, form the standard service context in which people with early psychosis are treated. This study aimed to explore the perspectives of mental health practitioners on loneliness in early psychosis including the extent of loneliness in this client group, drivers of loneliness and what might be done to help.

## Methods

We used the Consolidation Criteria for Reporting Qualitative Research (COREQ) to report the methods and results of the current study [32] (see Additional file 1).

### Ethics

The study was approved by the UCL Research Ethics Committee (ref: 6711/001) and the Health Research Authority (ref: 16/0227) before the commencement of data collection. Written consent was obtained from all participants.

### Setting

The study took place in four EISs in three London Mental Health Trusts, which cover socially and ethnically diverse inner and outer London boroughs. EISs are multidisciplinary community mental health teams offering up to 3 years support to people aged 14 to 65 following first episode psychosis [33].

### Participants

Purposive sampling was employed to capture a wide range of perspectives relating to the research question. We prioritised recruiting staff members from diverse professional groups with varied age, ethnic background and work experience. Mental health practitioners working in a clinical role (with a caseload or providing any type of direct patient care) at any EIS within the three participating London Mental Health Trusts were eligible for inclusion in the study. Exclusion criteria were working in a non-clinical role. Following guidance on sample sizes in qualitative research, we considered that a sample size of 15 participants would be sufficient to reach thematic saturation [34]. Data were reviewed at this stage with further interviews considered if new themes were still being identified from interviews.

### Procedures

Two researchers (TS, SJ) presented the study at team meetings in the eligible EISs. Staff members interested in the study were given the participant information sheet and gave their contact details to the researcher. Then, the lead researcher (TS) contacted the potential participants, discussed briefly the study, and arranged a time to meet with those who expressed a willingness to consider participation further. The composition of the sample was reviewed during the recruitment period to ensure a diverse sample. A minimum of 48 h between the study’s first contact and obtaining written informed consent was allowed. Socio-demographic data were recorded when informed consent was obtained (see Additional file 2). Out of twenty-two potential participants initially identified, twenty were finally recruited. One potential participant was excluded due to lack of direct patient care, and one refused participation for reasons that were not disclosed to the researcher. All participants were recruited from December 2016 to April 2017.

### Data collection

A qualitative interview guide was developed by the research team drawing on existing literature and early pilot work with two EIS practitioners. It covered four principal areas in relation to people in the early stages of psychosis: extent of loneliness; causes of loneliness; impact of loneliness; existing and potential interventions for loneliness (see Additional file 3). All interviews were conducted at the participants’ workplace, lasted approximately 1 h, and were audio recorded. During each interview only the researcher and the participant were present. Interviews were transcribed verbatim and transcripts were anonymized.

### Analysis

Interview transcripts were analysed using thematic analysis [35], supported by computer software (NVivo 11). All interview transcripts were analysed by one researcher (TS). A second researcher (JW) coded two interviews separately, and subsequently discussed the initial coding frame with the primary analyst. Other members of the research team reviewed emerging thematic ideas during the analytic process. The analysis used a combination of inductive and deductive processes, and followed a circular process of reading, coding and reflection to capture repeated and underlying patterns of data.

### Research team and Positionality

Researchers were mindful of how their own positioning and background might have influenced the collection and analysis of the data, and of the need to provide a transparent account of this to aid reader interpretation [36]. One researcher (TS) conducted all the interviews and led the analysis. TS is a mental health researcher with a psychology background. All other members of the team were academic mental health researchers with backgrounds in social care, social psychology and social psychiatry. JW, BLE and SJ have track records of research in loneliness. SJ is a psychiatrist who works clinically in early intervention for psychosis, and her observations in this setting contributed to her interest in this study.

## Results

### Sample characteristics

We interviewed 20 EIS mental health practitioners. The sample was demographically varied, comprising 60% women, 45% from non-White British ethnic groups, and participants from all adult age brackets, from 18 to 25 through to over 55 years old. Professional backgrounds were diverse, as intended, including psychiatrists, clinical psychologists, mental health nurses, social workers, occupational therapists, non-qualified staff and a CBT therapist. The demographic and professional characteristics of the participants are summarised in Table 1.

**Table 1:** Demographic and Professional Characteristics of the participants

Characteristic	Participants (***N*** = 20)
**Gender – N (%)**	
Male	8 (40)
Female	12 (60)
**Age group – N (%)**	
18–24	2 (10)
25–35	6 (30)
35–44	7 (35)
45–54	3 (15)
55–65+	2 (10)
**Ethnicity – N (%)**	
White British	11 (55)
White other	4 (20)
Black African	2 (10)
Black Caribbean	1 (5)
Asian Indian	1 (5)
Other	1 (5)
**Occupation – N (%)**	
Consultant Psychiatrist	2 (10)
Other Psychiatrist	3 (15)
Clinical Psychologist	2 (10)
Mental Health Nurse	5 (25)
Social Worker	2 (10)
Occupational Therapist	2 (10)
Non-qualified staff	3 (15)
Other (CBT Therapist)	1 (5)
**Years worked in Mental Health Services - Mean (SD)**	12.33 (10.34)
**Years worked at current EIS - Mean (SD)**	2.58 (2.61)
**Form of Employment - N (%)**	
Full-time	17 (85)
Part-time	3 (15)

Eighteen participants reported working in adult EISs providing care to service users with first episode psychosis over 18 years old. Two participants were working with service users aged 14 to 21 as one EIS team had a child and adolescent mental health team aspect embedded to it. Half of the participants were working as care coordinators (i.e. key workers planning and coordinating support for a caseload of service users).

### Qualitative findings

#### Overview

Qualitative findings are presented below according to three main areas of discussion in interviews: 1) How loneliness manifests itself among EIS clients; 2) Reasons why EIS clients are lonely and its effects on them; and 3) Potential ways of alleviating loneliness among EIS clients.

##### Manifestations of loneliness in EIS clients: social isolation and disconnection

Practitioners believed that most EIS clients experience loneliness. They reported that loneliness among clients is mostly expressed indirectly, and only a few openly speak about it.

*“They [service users] say, well, I’ve got nobody, I’m alone … They won’t come out and say, I’m lonely, but they come and say, I have nobody, nobody cares about me. Even if you ask about a neighbour, nobody there … don’t know anybody.” (Mental Health Nurse, Team A).*

Practitioners felt that EIS clients expressed their loneliness in two main ways. Firstly, most participants perceived socially isolated clients as lonely. They reported that many clients were socially withdrawn, spending most of their time at home without sources of connection:

*“And then you can see in their eyes that they’re lonely, when you ask them, how do you spend your day? Then they will say, mostly staying at home, indoors, maybe watching TV. Not interacting with anybody, living an isolated life, or just playing games.” (Specialist Psychiatrist, Team D).*

Some of the practitioners reported having clients whose isolation was such that their main social contacts were meetings with mental health professionals:

“*… I think there’s a big part of the population we look after that perhaps see just us. And I don’t think they may have other meaningful contact with other professionals or anyone else …*” *(Consultant Psychiatrist, Team B).*

Feeling different and disconnected emerged as another way that practitioners felt their clients expressed loneliness. They described how clients feel that they do not fit in and often avoid sharing important experiences with other people. This results in feelings of disconnectedness that hinder the development, and continuation of meaningful relationships.

*“I mean, the patient had friends, but there was definitely a disconnect in terms of emotional support … that she kind of felt like having to kind of live a secret life in front of her friends … Kind of this is me, I’m fine, but really, she’s experiencing these really horrible voices saying certain things.” (Social Worker, Team C).*

Some respondents also highlighted the importance of exploring whether clients seen as lonely by practitioners in fact experience loneliness.

*“And you might look at someone and it’s, oh my God, they spend five, six days a week on their own … They must be lonely. And I’ve had situations where people have said to me, I’m not lonely at all, really … So, I guess, number one, it needs to be identified as the service user’s perspective, that they feel lonely.” (CBT Therapist, Team D).*

##### Connections between loneliness and psychosis: symptoms, stigma and negative sense of self

Participants saw their clients as falling into two groups: those for whom loneliness emerged following the first episode of psychosis, and those for whom it was already a difficulty. In the first group, loneliness was seen as a by-product of psychosis. In the second group, practitioners linked premorbid loneliness to the lack of strong social networks and history of trauma. They believed that loneliness breeds loneliness with this second group being more vulnerable to loneliness after the onset of psychosis.

*“… is about how well connected they are before their illness … that’s where if they didn’t have strong friendships to start with and then they got ill and their illness kind of took them into hospital for a period of time and took them away from their normal life so to speak that’s when they then come back to that and back into community. That’s when they really talk about kind of loneliness.” (Mental Health Nurse, Team D).*

In both client groups, three features were identified as having mutually reinforcing relationships with loneliness: symptoms (psychotic and affective symptoms), stigma, and negative sense of self.

##### Symptoms

Participants reported observing a bidirectional interplay between loneliness and mental health symptoms. Feelings of depression and anxiety, positive and negative symptoms of psychosis, and suicidal ideation were related to loneliness. Some participants suggested that service users with more affective symptoms tended to be lonelier, and lonely service users presented more feelings of sadness and low mood.

*“...[loneliness] is going to affect their mood and the way that they [service users] manage things, so they might feel lower in mood as an effect of being lonely, they might feel less able to deal with things and manage things, because they feel unsupported …” (Occupational Therapist, Team C).*

Several participants also thought that the experience of psychotic symptoms could initiate or maintain loneliness. It was even suggested that prolonged feelings of loneliness could trigger a first episode or lead to a relapse:

*“… a good example is a patient who recovered and who has been discharged, but he felt lonely for years, and then you discharge the patient without taking into consideration that that loneliness was also a trigger for his illness … so, eventually if they continue to feel lonely … then obviously there is a risk factor … which could be a contributing factor for a possible future relapse.” (Specialist Psychiatrist, Team A).*

Paranoia in particular was seen as having a reciprocal relationship with loneliness and social anxiety: feeling paranoid increased loneliness and social anxiety, while loneliness also fed into paranoia. Although there was a consensus among participants regarding the negative impact of symptoms in loneliness and vice versa, one participant described auditory hallucinations at times counteracting some clients’ feelings of loneliness:

*“… Because clients can develop a relationship with their voices which can sometimes fill the gap of loneliness. Sometimes people say, actually, I like my voices because it’s like having friends around me, and they can have the connection with the voices themselves.” (Clinical Psychologist, Team C).*

##### Stigma and negative sense of self

Most of the participants thought that stigma associated with mental illness caused and/or maintained loneliness for their clients. They described how clients often became gradually estranged from friends and family after their first psychotic episode. This stigma was attributed to the media, and to the lack of public awareness around mental health problems.

*“I have one service user who feels she can’t talk to her husband because whenever she talks to her husband, he throws the fact that she has a mental problem or a disability back into her face, so she feels reluctant to share with him any information. And obviously you’re in a relationship, and so being unable to share that information does make that person feel quite lonely.” (Non-Qualified staff, Team A).*

Participants also talked about the reciprocal relationship between negative view of self and loneliness. The internalisation of mental illness stigma into people’s identity fosters low self-esteem and feelings of worthlessness that can in turn result in social withdrawal and feelings of loneliness:

*“He [service user] is probably been quite lonely for quite some time … and he talks to himself, and people in his community sort of berate him for that … yes, he feels quite judged by his community because he’s not achieved … So, he wouldn’t go out until late at night, so his people wouldn’t see him.” (Mental Health Nurse, Team D).*

The two clinical psychologists described social comparisons as a pathway to loneliness, leading to avoidance and a further loss of confidence reinforcing the negative evaluation of one’s self.

*“He was withdrawing because he was comparing himself with how he used to be, and with others. And so, therefore it was easier to just opt out and avoid … so then you’d get into the cycle of withdrawing, losing your confidence, and then becoming lonely.” (Clinical Psychologist, Team A).*

#### Service responses to loneliness

##### Existing interventions

Participants reported that their EIS teams did not offer targeted loneliness interventions. However, they described how existing interventions or initiatives with a different primary objective could be beneficial for reducing loneliness. These included social groups, psychological interventions, vocational support, and religious groups provided by the EISs and/or the local boroughs.

The most cited intervention for addressing loneliness was social groups provided by the EIS or the local boroughs. These included a wide range of activities such as going to the cinema, going for coffee, playing board games, or physical exercise. Reported patient benefits varied, and seemed to be related to the severity of symptoms, the level of motivation, and engagement with services.

*“...the social group hasn’t been helpful for the lady that’s at home all the time because she’s unable to go out. But certainly, for the other gentleman that’s very lonely and doesn’t have any friends or family, he’s found the groups extremely helpful and he tends to, when he’s well enough he tends to come to the groups and contributes and socializes that way.” (Social Worker, Team A).*

Despite the EIS model’s aspiration to support integration in mainstream provision as far as possible, stigma was seen as a barrier in engaging people with groups not related to mental health services:

*“...If we refer into the cooking groups run by the local borough, they want a care coordinator to attend the session with them, because they have some misguided ideas about mentally ill people using kitchen equipment … so that’s quite … disheartening, from our point of view.” (Mental Health Nurse, Team D).*

Psychological interventions were also seen as having secondary benefits for loneliness. Psychological interventions included therapeutic groups, family therapy and individual therapy, with the majority of them being offered by the EIS. The therapeutic groups consisted mostly of CBT-based psychoeducation groups, but also music and art groups. There was consensus among respondents that, despite limited availability, individual talking therapy could be beneficial in reducing feelings of loneliness by targeting negative self-schemata:

*“… I personally think that more active psychology could help, psychology in any form … it has the capacity … to change the way they [service users] perceive themselves, and their social contacts …” (Non-Qualified staff, Team B).*

Help with employment, including voluntary work and skills-based resources, as well as in obtaining employment, was seen as another indirect way of addressing loneliness. Religious communities were also cited by a few as helpful in reconnecting people with religious affiliations back to their communities:

*“… they’re often quite tight knit church communities, who took very seriously the idea of visiting people in hospital and looking after people. And a lot people especially from African demographic spend a lot of free time in church …*.” *(Mental Health Nurse, Team B).*

#### What else could be done?

Accounting for patient’s stage of recovery was seen as critical for the effectiveness of any potential loneliness intervention. Respondents emphasised that an individualised approach would be needed, with each client’s unique social circumstances, stage of recovery, interests, values and current symptoms shaping their needs. As a result, a single generic intervention for loneliness might fail to help them. Views on what provision could usefully be developed varied, but with many participants emphasising the need to consult clients:

*“And the solution [to loneliness] needs to be driven by people, and I guess it’s very useful to ask about it … from the simple things like why don’t we have a badminton group...And maybe if we listen to people we’ll set up cafés next, or we’ll do other things that perhaps would be a lot more useful in addressing loneliness.” (Consultant Psychiatrist, team B).*

Some respondents viewed loneliness as a broader societal problem and not specifically related to mental illness. They believed that it should be tackled at multiple levels including the mental health services, service users, families, local communities and society as whole.

*“It’s everybody’s responsibility to reduce loneliness … there’s something around certain events that happen in London that I think people feel a sense of community … So, there’s something at that level, but also within a family, within a group of friends. So, this is the café, this is the pub, the landlords …” (Clinical Psychologist, Team A).*

Participants talked about increasing opportunities for social interaction and linking people back to their communities as ways to alleviate loneliness. The usefulness of fighting stigma alongside signposting people to community groups without a mental health focus were reported as potentially beneficial for loneliness:

*“Something with a social aspect would be helpful for loneliness … but something that’s not just mental health specific. Because I just think that marginalizes people … like, I had a conversation with one of my clients about joining a book club. So, stuff like that.” (Mental Health Nurse, Team D).*

On the service level, participants suggested that all EIS staff should be mindful that loneliness is a common feature in psychosis and create space for people to able to talk about it. A holistic approach for tackling loneliness was seen as key with practitioners describing the importance of seeing service users as complete social beings. They talked about spending more time with clients, having meetings without a mental health focus and exploring their interests. A concern expressed by some respondents was that through forging a good working alliance, they may become the primary social contact for some isolated service users:

*“... Like, I see him [the service user] once every other week. And he said to the psychologist, when asked, what do you do with your time? He said that he hangs out with me. And I just thought that was really sad, because... You know, when I’m paid to, you know....” (Occupational Therapist, Team D).*

The use of online apps and forums was seen as a promising tool for reducing loneliness by a few participants. Lack of romantic relationships was also perceived as contributing to loneliness, and a few practitioners thought there might be scope for EISs to take a more active role in helping clients to meet this need.

## Discussion

To our knowledge, the present study is the first to provide an in-depth exploration of loneliness in early psychosis from the perspective of a key stakeholder group. As a result, it fills a gap in the research literature, helping to develop the knowledge base around loneliness in early psychosis and potential ways of addressing it. We found that EIS practitioners viewed their clients as lonely but loneliness was rarely spoken in EIS routine clinical practice reflecting previous research about the stigma of loneliness and difficulties in self-disclosure [1, 37, 38]. Practitioners understood that service users were lonely when they were socially isolated and expressed feelings of disconnectedness. These findings are consistent with previous research suggesting the social and emotional dimensions of loneliness [39], as well as its overlap with objective social isolation [27]. Some participants highlighted the importance of identifying loneliness from the perspective of service users reflecting the idea that loneliness is related but also distinct from objective social support [2]. There is a need for future research to explore loneliness from the perspectives of other key stakeholder groups, including service users and carers.

According to practitioners’ accounts, feelings of loneliness can emerge after the first episode psychosis, or they can precede it. In the second instance, prolonged feelings of loneliness alongside trauma and life adversities were seen as a trigger for the first episode. This is an interesting findings that warrants further research and has been proposed in psychoanalytic literature whereby psychosis was partly viewed as a defense to loneliness and agony [40]. Our analysis suggests that EIS service users with reduced social networks might be more prone to loneliness, but there is preliminary evidence indicating that the relationship between loneliness and social network may be different in people with psychosis compared to other diagnostic groups [41]. Thus, more research is needed to explore the social cognitions, symptoms and social factors, which contribute to loneliness for people with psychosis, and whether/to what extent these may differ from other mental health groups.

Practitioners believed that loneliness is a complex multi-faceted phenomenon that has reciprocal relationships with symptoms, especially affective, stigma and maladaptive cognitions. These findings are consistent with previous research suggesting a series of possible mechanisms through which loneliness links to psychosis [23, 24]. Participants recognized a bidirectional effect between loneliness and symptoms suggesting that both depression and paranoia can lead to loneliness. Our findings also concur with other research [42], in suggesting that auditory hallucinations can counteract feelings of loneliness for some service users. Practitioners’ perspectives further suggested that stigma attached to psychosis can lead clients to withdraw from social life, which can in turn increase feelings of loneliness, with previous literature suggesting stigmatisation as one of the main causes of loneliness among people with mental health problems [43]. Our results also add on existing research suggesting the importance of negative sense of self in the initiation and perpetuation of loneliness [23].

Moreover, our findings highlight that addressing loneliness directly was not being attempted within the EISs that we studied. Participants reported that there are a number of existing interventions with a different primary objective offered in the EISs, and in the community, that can be beneficial for loneliness. These included social groups, psychological interventions, vocational support, and religious communities. Even in the absence of an evidence-based intervention directly targeting loneliness, clinicians in the EISs can make use of the existing services and signpost their clients accordingly. With sufficient resource allocation, greater access to individual talking therapies is required. Respondents in the study indicated that staff members could do more things in a service level to alleviate loneliness. Feasible and useful actions included: spending time with clients without a mental health focus, asking clients what else the services can offer to help them with loneliness, facilitating clients’ suggestions, and exploring online activities that can reduce feelings of loneliness. However, there has been a reduction in the number of staff contacts per patient in community mental health teams in recent years in England [44]. Literature further suggests that mental health staff often acknowledge and provide less support with developing social connections than service users would like due to system level barriers [45], and social connections are not a focus of the nationally audited standards for EIP teams in England [33]. Adequate resourcing for EIP teams and policy prioritisation of support with social relationships may be required to enable EIS practitioners to have capacity to undertake targeted individual work to address loneliness.

According to the participant accounts, addressing loneliness in early psychosis is not something clinical services can easily take on by themselves, but more readily as part of a multilevel intervention, that targets stigma too [46]. Consulting clients on what can be done, and facilitating their suggestions was considered necessary to inform effective and acceptable loneliness interventions. Participants recommended that increasing opportunities for social interactions and linking people back to their communities are potentially promising in alleviating loneliness for service users with early psychosis. These views are not fully in line with preliminary evidence indicating that addressing maladaptive cognitions might be more beneficial than increasing social participation for reducing feelings of loneliness [20, 29, 30]. This discrepancy warrants further exploration as it can inform future treatment targets for loneliness in early psychosis treatment settings. This inconsistency might also reflect the study’s sample composition whereby around half of the participants were care-coordinators and only two respondents were in psychology positions. A large quantitative survey of staff views might be appropriate to examine and compare different professional group’s views and experiences. Finally, digital technology was also seen as having potential for the alleviation of loneliness, with scope for online lives to be addressed more than at present in the work of EIS. Lastly, the need for intimate relationships should be considered in the development of loneliness reduction interventions.

Our findings have important implications for future intervention development. Practitioners valued social participation interventions without a mental health focus and suggested a co-produced individualised approach to intervention development. Therefore, wider community approaches [28] that take an individualised approach to linking service users to social groups or activities that fit with their strengths, interests and social identity may be of high interest for future research. Groups for Health (G4H) and Connecting People Intervention (CPI) are promising interventions with other client groups [47, 48]. Our study further added on the existing literature suggesting the significant role of symptoms (especially affective), stigma and negative sense-of self on the loneliness pathway. Future research can evaluate whether these could act as targets for loneliness interventions.

### Strengths and limitations

Interviewing a substantial number of staff with varying demographic and professional characteristics increases confidence that the study results captured a diverse range of practitioner perspectives. Although some professional groups might view things differently from others, our study was not designed, and our sample was not sufficient to explicitly compare different staff groups’ perspectives. This may be a useful focus for future research. Involving two researchers in the development of the initial coding frame enhanced the validity of the study. However, all interviews were conducted by only one researcher, which may have biased the way interviews were conducted (e.g., how interview prompts were used) and resultant participant reports. Furthermore, the study involved just a small number of EIS teams all in the London area, so results may not generalise. Finally, while our analysis team included people from a range of professional disciplines, and one current EIS clinician, involving other relevant stakeholder perspectives, such as those of service users, might have strengthened the analysis.

## Conclusions

We found that EIS practitioners see service users with early psychosis as lonely, but loneliness is rarely spoken about in routine clinical practice. Although a targeted strategy for tackling loneliness is lacking, EIS practitioners can make use of existing services and interventions with a different primary objective. A co-produced, multilevel, individualised intervention that will aim to reconnect people to their communities and create opportunities for social interaction without a mental health focus might be promising in alleviating loneliness among people with early psychosis. Symptoms, stigma and negative sense of self were believed to interplay with loneliness and can act as targets for future interventions. There is a need for high quality research to assess the effectiveness of current therapeutic practices in reducing loneliness, and to develop effective interventions that specifically target loneliness.

## Supplementary Information

**Additional file 1.** Consolidated criteria for reporting qualitative studies (COREQ): 32-item checklist.

**Additional file 2.** Participant Background Information.

**Additional file 3.** Semi-Structured Interview Topic Guide V1.1 15/01/17.

## Data Availability

The datasets generated and/or analysed during the current study are not publicly available due to lack of consent from participants to share full transcripts but are available from the corresponding author on reasonable request.

## Abbreviations

EIS: Early Intervention Service
G4H: Groups for Health
CPI: Connecting People Intervention
