# Supplementary material for: Loneliness in early psychosis: a qualitative study exploring the views of mental health practitioners in early intervention services
Source: BMC Psychiatry. 2021 Mar 6;21:134. doi: 10.1186/s12888-021-03138-w (PMC7937295; doi:10.1186/s12888-021-03138-w)
Supplement: Supplementary file 2 — Additional file 2. Participant Background Information. [file 12888_2021_3138_MOESM2_ESM.docx]

**Participant Background Information**

**Participant ID:**

**Service:**

**Date:**

**Age group:**

18-24

25-34

35-44

45-54

55-64

65+

**Gender:**

**1** 1 Male


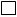
 2 Female

**Ethnic background:**

| 1 1 White British    2 White Irish    3 White Other    Black/Black British  4 Caribbean    5 African    6 Black Other    Asian/Asian British  7 Indian    8 Pakistani    9 Bangladeshi    10 Sri-Lankan    11 Asian Other | Mixed  12 White/Black Caribbean    13 White/Black African    14 White/Asian    15 Other mixed    Chinese or other  16 Chinese    17 Other ethnic group |
| --- | --- |

**Name of the Mental Health Team that you currently work:**  _______ _

**How long have you worked in Mental Health Services?** ___Years ___ Months

(Please include any time spent as a student/trainee)

**How long have you worked in this Early Intervention Team?**  ___Years _Months

**Are you currently working full-time or part-time?**

Full-Time

Part-Time

**Which of the following describes your occupation?**

Psychiatrist

Clinical Psychologist

Social Worker

Team Manager

Mental Health Nurse

Occupational Therapist

Clinicians who are not qualified professionals (e.g. support worker, graduate mental health worker, psychology assistant, nursing assistant, assistant practitioner etc.)


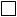
 Other, please specify _____________

**Level of seniority (e.g. senior, junior)**
