# Supplementary material for: Loneliness in early psychosis: a qualitative study exploring the views of mental health practitioners in early intervention services
Source: BMC Psychiatry. 2021 Mar 6;21:134. doi: 10.1186/s12888-021-03138-w (PMC7937295; doi:10.1186/s12888-021-03138-w)
Supplement: Supplementary file 3 — Additional file 3. Semi-Structured Interview Topic Guide V1.1 15/01/17. [file 12888_2021_3138_MOESM3_ESM.docx]

**Semi-Structured Interview Topic Guide V1.1 15/01/17**

**Study:** A qualitative study exploring the views of mental health practitioners on loneliness among service users with early psychosis

**Participant ID:**

**Date of the Interview:**

**Introduction**

1. Explain the nature and purpose of the study
2. Introduce Dictaphone
3. Highlight confidentiality

**Introductory Questions**

- How do you understand loneliness**? (**Before asking the main questions make sure both interviewer and interviewee conceptualise loneliness similarly)
- Which main client group do you currently work with?

**Main Questions**

1. To what extent do you feel that your clients experience loneliness?

**Probes**

- What makes you think they are lonely?
- Is it a problem for just a few clients or a lot of them?
- How loneliness is expressed among clients that are lonely?

1. Thinking of your caseload, are there any certain groups of clients who may be more prone to loneliness?

**Prompts:** specific diagnostic categories, specific symptoms, age, culture, employment, comorbidities

1. Why do you think some of your clients get lonely?

**Probes**: mental health, physical health, social factors?

1. Which do you think are the factors that maintain loneliness for your clients?

Probes

- Could you describe a case?

1. How do you think loneliness affects your clients?

**Probes**

- Can you think of any other things that it can affect?

1. For service users who seem lonely how do you tend to respond?

**Probes**

- Do you feel that your response is informed by any training or previous experience?
- Do you think that the existing resources in your team affect your response? In which way?

1. Does your team or the community have any existing ways of helping that might be beneficial for service users who are lonely?

**If yes, probe**

- Could you tell me a little bit more about it?
- Do service users engage with it?
- How effective do you think it is in helping people feel less loneliness?
- Anything that prevents this being used as a way of helping lonely people?
- Anything that helps?

**If no, probe**

- Why not?

(**Prompts:** e.g. is it a matter of limited resources? Is it because loneliness is an unacknowledged problem?)

1. In your opinion whose role is it to try to reduce loneliness among service users with psychosis?

**Prompts:** Mental health services, community

1. Based on your knowledge and experience in mental health what interventions would you most like to be able to offer or refer your clients to for the problem of loneliness?

**Probes**

- Could you tell me a little bit more about it?
- Do service users engage with it?
- How effective do you think it is in helping people feel less loneliness?
- Anything that prevents this being used as a way of helping lonely people?
- Anything that helps?

1. Do you have anything more to add to any of the topics we've covered or anything else that you think is relevant to what we’ve talked about?

**Thank you!**
